# Photobiostimulation conjugated with stem cells or their secretome for temporomandibular joint arthritis in a rat model

**DOI:** 10.1186/s12903-023-03466-1

**Published:** 2023-10-05

**Authors:** Rana El-Qashty, Osama A. Elkashty, Eman Hany

**Affiliations:** 1 Oral Biology Department, Faculty of Dentistry, Mansoura University, Mansoura, Egypt; 2 Oral Pathology Department, Faculty of Dentistry, Mansoura University, Mansoura, Egypt

**Keywords:** Joint diseases, Low level laser therapy, Adipose-derived mesenchymal stem cells, Cell free therapy, Tumor necrosis factor, Toluidine blue

## Abstract

**Background:**

Temporomandibular joint (TMJ) arthritis is a debilitating, challenging condition and different methods have been implicated for its treatment. This study aimed to test the therapeutic potentials of low-level laser therapy (LLLT) associated with adipose derived stem cells (ADSC) or their derived secretome on a murine model induced arthritis.

**Methods:**

Forty eight rats were divided into four groups where group I was the sham control, the rest of animals were subjected to arthritis induction using complete Freund’s adjuvant, then divided as follows: group II received phosphate buffered saline (PBS) intraarticular injection and irradiation of 0 j/cm2, group III received ADSCs derived secretome and irradiation of 38 j/cm2, and group IV received ADSCs and irradiation of 38 j/cm2 as well. One and three weeks after treatment, animals were euthanized, and paraffin blocks were processed for histological assessment by hematoxylin and eosin stain with histomorphometrical analysis. Histochemical evaluation of joint proteoglycan content was performed through toluidine blue stain, and immunohistochemical staining by the proinflammatory marker tumor necrosis factor-α (TNF-α) was performed followed by the relevant statistical tests.

**Results:**

The arthritis group showed histological signs of joint injury including cartilage atrophy, articular disc fibrosis, irregular osteochondral interface, and condylar bone resorption together with high inflammatory reaction and defective proteoglycan content. In contrast, the treated groups III and IV showed much restoration of the joint structure with normal cartilage and disc thickness. The inflammation process was significantly suppressed especially after three weeks as confirmed by the significant reduction in TNF-α positive immunostaining compared to the arthritic group, and the cartilage proteoglycan content also showed significant increase relative to the arthritic group. However, no significant difference between the results of the two treated groups was detected.

**Conclusion:**

LLLT conjugated with ADSCs or ADSCs derived secretome can efficiently enhance the healing of arthritic TMJs. Stem cell secretome can be applied as a safe, potent therapy. However, further investigations are required to unravel its mechanism of action and pave its way as a safe, novel, cell free therapy.

**Graphical Abstract:**

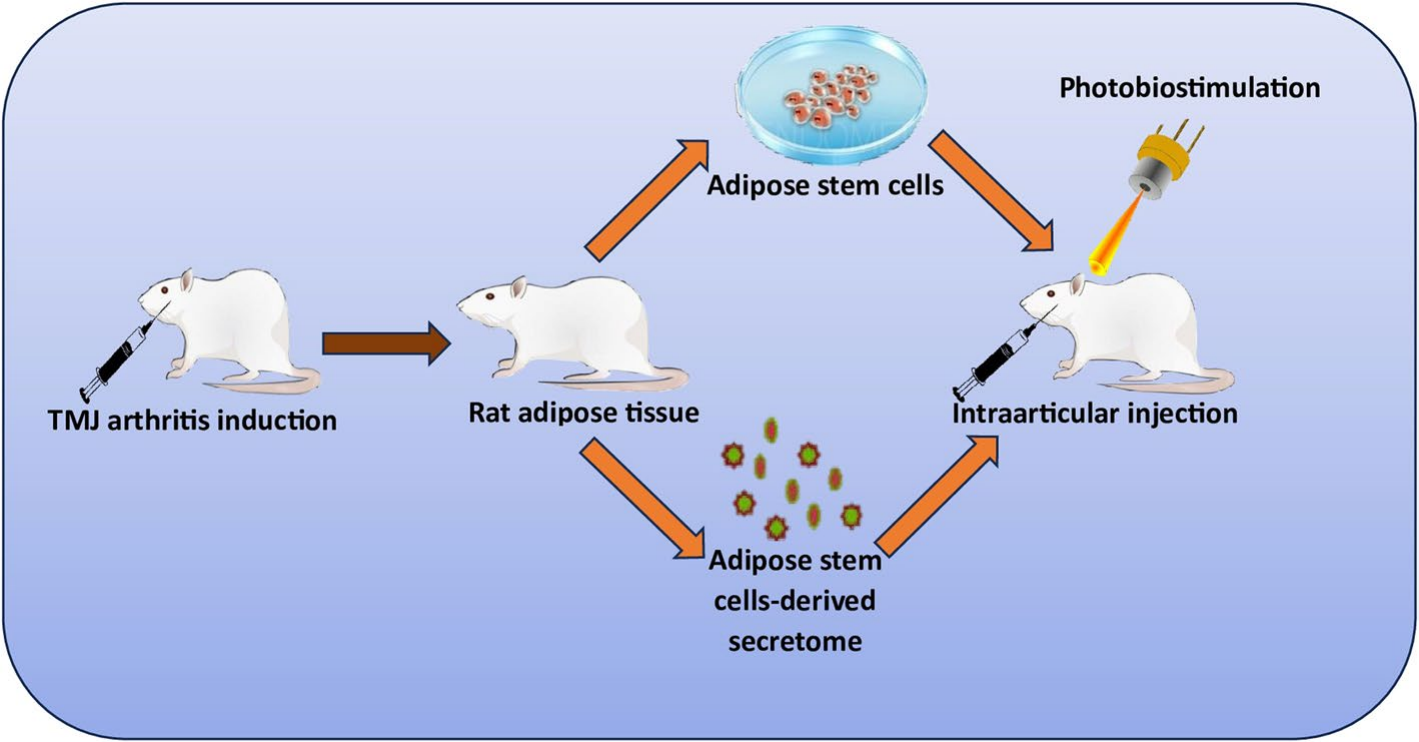

## Background

Temporomandibular joint (TMJ) is a unique joint in the human body due to the complexity of its structure and movements [1]. The articulation system that constitutes the TMJ includes the articular disc, jaw muscles and occlusion. It articulates the upper and lower jaws where teeth regulate the contact between the jaws while muscles move the mandible. TMJ is a bilateral joint where any stimulus affecting one joint or any part of the articulation system would hazard the rest of the system [2].

TMJ arthritis is a multifactorial condition that affects the TMJ and the associated masticatory muscles and may cause many symptoms including pain, difficulty in speech and feeding in addition to altered facial appearance which may affect the quality of life. Diagnosis and management of TMJ arthritis represents a great challenge [3].

Low-level laser therapy (LLLT) has been widely used in recent years as a conservative method of treatment. It has been successfully implicated in different clinical applications such as the treatment of gummy smile [4], different surgical procedures [5], as well as myogenic TMJ disorders [6]. It has photobiostimulatory properties on tissues with no direct thermal effects [7]. It also has analgesic, anti-inflammatory, and regenerative effects. The bio-stimulator properties of LLLT are associated with increasing cellular metabolism, microcirculation and collagen synthesis as well as enhancing chemotaxis and blocking pro-inflammatory mediators [8]. Among the benefits of dental laser treatment is the selective and precise interaction with diseased tissues, without injuring the surrounding tissue or causing any significant complications [5]. LLLT has gained great acceptance among patients as it doesn’t cause any distress and is considered a minimally invasive procedure [4].

In the past decade, progress made in the development of stem cell-based therapies and tissue engineering has provided alternative methods to attenuate the disease symptoms and even replace the diseased tissue in the treatment of TMJ disorders [9]. Mesenchymal stem cells (MSCs) possess multilineage differentiation potential and have been applied in cell therapy for joint arthritis [10]. They have the ability to prevent pro-inflammatory cytokines release and to produce soluble anti-inflammatory factors influencing leukocyte accumulation in the joint fluid during inflammation. MSCs can also differentiate directly into chondrocytes leading to cartilage regeneration [11]. Among the various tissue sources of MSCs, adipose tissue emerged as an affluent and stable source of MSCs in terms of large quantity, high yield, ease of access, less invasive procedures and no ethical issues [12].

However, cell therapy has some limitations regarding the maintenance of cell viability, functions, possibility of carcinogenic transformation, immunogenic rejection, and other safety concerns. Moreover, therapeutic potential of stem cells is mainly attributed to the paracrine effect of the cells that secrete different factors into their media [11] where MSC secretome was found to have anti-inflammatory and anti-apoptotic effects [13]. So, stem cell secretome is becoming a promising novel cell free therapy for wound healing in regenerative medicine [11].

Tumor necrosis factor alpha (TNF-α) is a cytokine that has an important proinflammatory role in arthritis where together with other pro-inflammatory cytokines, TNF-α mediates the inflammatory response of joint chondrocytes, synovial fibroblasts, and osteoclasts that secrete tissue-damaging enzymes such as the matrix metalloproteinases [14]. TNF-α has been used as an inflammatory marker in arthritis and was correlated to the severity of the disease [15].

To date, only limited studies have been conducted on the TMJ detecting the effect of the combinatory treatment of stem cells or their secretome along with LLLT in arthritic models. So, this study aimed to compare the regenerative effect of low-level laser therapy in conjunction with adipose derived stem cells (ADSCs) or adipose derived stem cell-secretome in a murine temporomandibular joint arthritis model.

## Materials and methods

### Animals

Sample size was calculated using G*power version 3.1.9.4 based on the mean thickness of the TMJ articular disc among the studied groups retrieving the control and arthritis groups from a previous study [16]. With 2.715 as effect size, 2-tailed test, 0.05 α error and 90.0% power, the calculated total sample size was at least five in each group. Six rats were used in each group to compensate for the drop rate.

Forty-eight male, pathogen free, Sprague Dawley rats, of weight 250–300 gm were selected. Animals were housed in Mansoura experimental research center (MERC), Mansoura University, Mansoura, Egypt. Six rats per cage were kept in a light-controlled room with a 12h light–dark cycle, 65–70% relative humidity and 22 ºC temperature. Commercial diet and water were served to animals *ad-libitum*. They were acclimatized for two weeks preceding the experiment.

### Study design

This was an experimental, randomized, controlled study. Rats were randomly allocated into four groups (*n* = 12) using simple random sampling method as follows:

- *Group I (Sham control)*: rats received intraarticular injection of 50 μL of phosphate buffered saline (PBS) and after one week, they received another 0.1 ml PBS and were subjected to diode laser application of 0 J/cm^2^.

The remaining rats were subjected to arthritis induction by single intraarticular injection of 50 μL of complete Freund’s adjuvant (CFA) (cat. #F 5881, Sigma Aldrich, St. Louis, Missouri, United States) [10] in the left TMJs only not to hinder the chewing ability and feeding of animals. One week later, rats were divided into three groups:

- *Group II (Arthritis)*: rats were subjected to intra-articular injection of 0.1 ml PBS, followed by laser application of 0 J/cm^2^.
- *Group III (Secretome / LLLT)*: rats were subjected to intra-articular injection of secretome (0.1 ml), followed by diode laser application of 38 J/cm^2^.
- *Group IV (ADSCs / LLLT)*: rats were subjected to intra-articular injection of ADSCs (1 × 10^6^ cells) suspended in 0.1 ml PBS [17], followed by diode laser application of 38 J/cm^2^.

One week and three weeks from the onset of LLLT, six rats of each group were euthanized by intraperitoneal thiopental overdose.

### Adipose Derived Stem Cells (ADSCs) isolation

For ADSCs isolation, six, healthy, Sprague Dawley rats weighing 200–250 g (5–6 months of age), were used following our previous protocol [18]. Adipose tissue was obtained from the supra-renal fats and extensively washed, minced into pieces, and then digested in 0.1% type I collagenase (cat. #SCR103, EMD Millipore Corp, Billerica, USA), for 1h at 37°C. Dulbecco’s modified eagle’s medium (DMEM) (cat. #L0066-500, BioWest, Nuaillé, France) culture media supplemented with 10% fetal bovine serum (FBS) (cat. #S1810-500, BioWest, Nuaillé, France) was then added to neutralize the action of collagenase enzyme. Cells were kept in an incubator and media changed every 2–3 days. An inverted microscope (Olympus, CKX41SF, Tokyo, Japan) was used to examine the cell cultures daily till reaching 80% confluence.

### Adipose derived stem cells characterization

For ADSCs characterization, cells at the third passage were incubated with the following primary antibodies: CD73 (BD Biosciences, cat. #551123), CD90 (BD Biosciences, cat. #551401), CD34 (R&D systems, cat. #AF6518-SP) and CD45 (BD Biosciences, cat. #561867). For purified antibodies, Fluorescein isothiocyanate (FITC, cat. #F143, Thermo Fisher Scientific, Massachusetts, USA), as the fluorophore, was added separately, then incubated for 30 min in the dark at 4 °C. Cells were then washed with PBS, centrifuged at 200xg for 5 min, and resuspended in PBS. BD Accuri C6 flowcytometer (BD Biosciences, USA) was used for flowcytometric analysis.

### Secretome preparation

Secretome was prepared from ADSCs at the third passage (12 × 10^6^ cells). After reaching 80 to 90% confluence, the ADSCs were washed three times with PBS, and the media were replaced with serum-free DMEM. Cells were incubated in the serum-free media for 48 h. The media were then collected and filtrated through a 0.2 μm filter to remove cellular debris and stored at -80°C until use [19].

### TMJ intra-articular injection technique

**Fig. 1:**
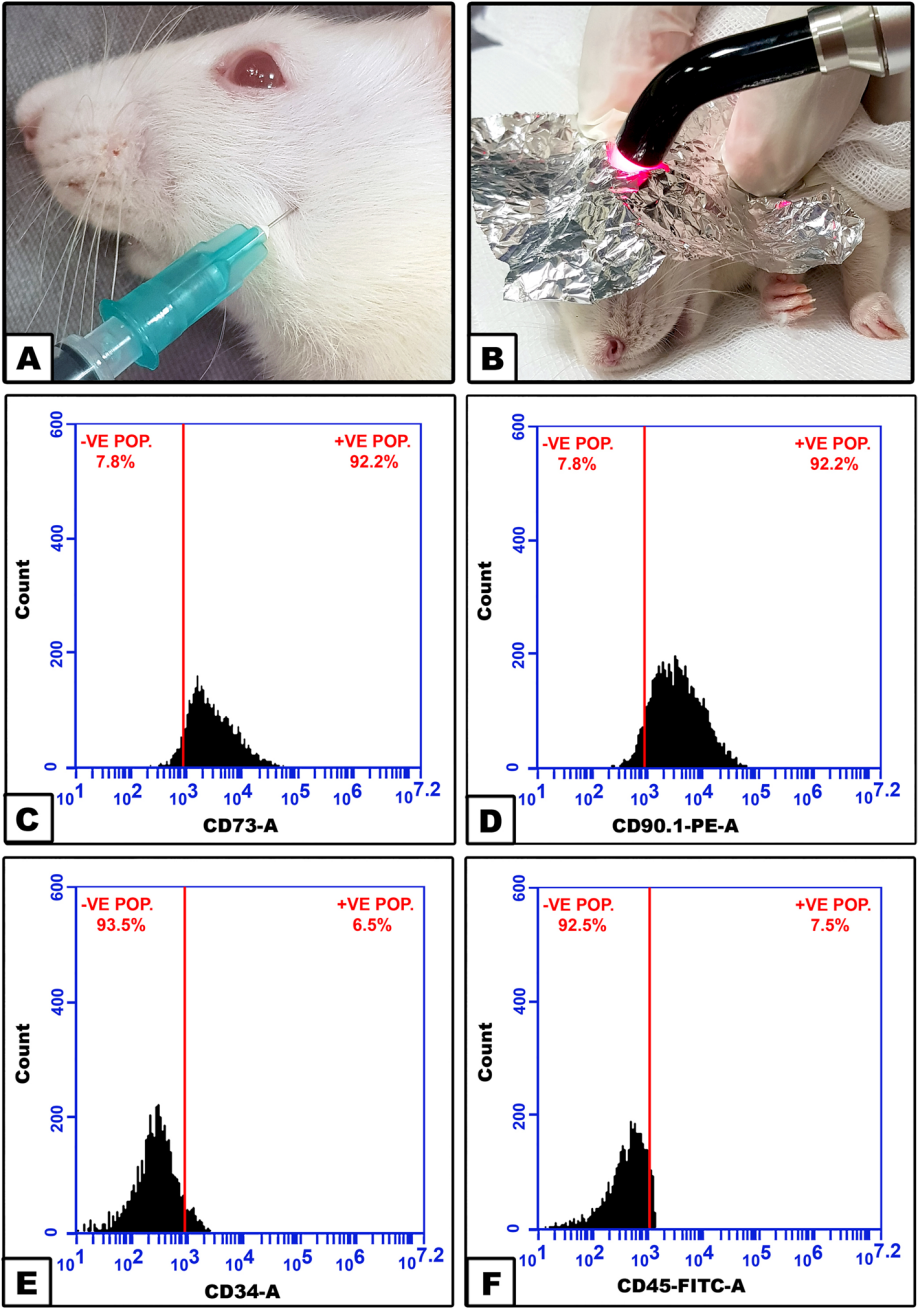
Photograph showing techniques of intraarticular injection (**A**) and photobiostimulation (**B**). ADSCs flowcytometric phenotypic characterization histograms for CD73 (**C**), CD90 (**D**), CD34 (**E**), CD45 (**F**)

Rats were anesthetized by halothane inhalation (4–5%) in a closed tube, then TMJ regions were shaved and wiped with Betadine. The TMJ area was palpated, and intra-articular injection was performed following our previous protocol [16]. PBS, CFA, ADSCs or secretome were injected according to the intervention of each group (Fig. 1A).

### Photobiostimulation

LLLT was performed following our previous methodology [16] using Cheese II medical Diode laser device (Wuhan, China) with the parameters illustrated in Table 1. Rats’ TMJs were located by palpation lateral to the outer canthus of the eye by 5–10 mm together with moving the mandible. After intra-articular injection, while animals were under anesthesia, areas around the TMJs were covered by aluminum foils to avoid injury to the animals’ eyes, and laser was applied perpendicular to the skin over the TMJs (Fig. 1B).

**Table 1:** Photobiostimulation parameters

Active medium	InGaAs (semiconductor)
Wavelength	980 nm
Tip diameter	1 cm
Irradiation mode	Continuous
Power	0.5 watt
Power density	0.64 watt/cm2
Energy	30 J
Energy density	38 J/ cm^2^
Duration	60 sec
Distance	0.5 cm
Irradiation	Every 48 hrs for 7 days (4 sessions)

### Histological examination

After euthanization, the TMJs were collected and fixed in 10% neutral buffered formalin for 24 h. Samples were then decalcified and dehydrated. Paraffin blocks were prepared and serial sections of 4–5 µm thickness were cut. Slides were stained by hematoxylin and eosin stain (H&E) for examination of histological structure, immuno-histochemical stains for tumor necrosis factor alpha (TNF-α) to assess the inflammatory process, and toluidine blue histochemical stain for detection of proteoglycans.

### Digital image analysis

A blind examiner was designated to examine and photograph the histological slides using a digital camera (ToupCam®, XCAM1080PHA, Russia) with a photo-adaptor (0.5X) mounted on a light microscope (Olympus®, CX22, Japan), using 10X objective lens. An Intel Core i7 based computer was used to analyze the images using Fiji ImageJ software (version 1.51r; NIH, Maryland, USA). Measurement calibration was performed, and the thickness of the articular disc and condylar cartilage was measured at three different locations in each slide: anteriorly, posteriorly and at the middle. Mean values were calculated.

The immunohistochemically stained slides were examined using the 40X objective lens and five sites per slide were photographed for digital analysis. Positive staining appeared as brown deposits and were assessed by calculating the percentage of the positive reaction area to the total area of each photomicrograph. Toluidine blue-positive staining total area at the TMJ cartilaginous zone was calculated to evaluate the proteoglycan content.

### Statistical analysis

Data was analyzed using GraphPad Prism 9 (GraphPad Software). Quantitative data was described using mean ± standard deviation for normally distributed data after testing normality using Shapiro–Wilk test. The significance of the obtained results was judged at the (0.05) level. Two Way ANOVA test was used to assess the combined effect of the two independent factors (intervention and time) on the articular disc thickness, condylar cartilage thickness, TNF-α expression and toluidine blue metachromism followed by Post Hoc Tukey test for pairwise comparison.

## Results

### Flow-cytometry characterization

After subjecting ADSCs to phenotypic cell surface marker analysis, the mesenchymal markers CD73 (92.2%) and CD90 (92.2%) were found to be highly positive while the hematopoietic markers CD34 (6.5%) and CD45 (7.5%) were negative (Fig. 1C-F).

### Hematoxylin and eosin staining

**Fig. 2:**
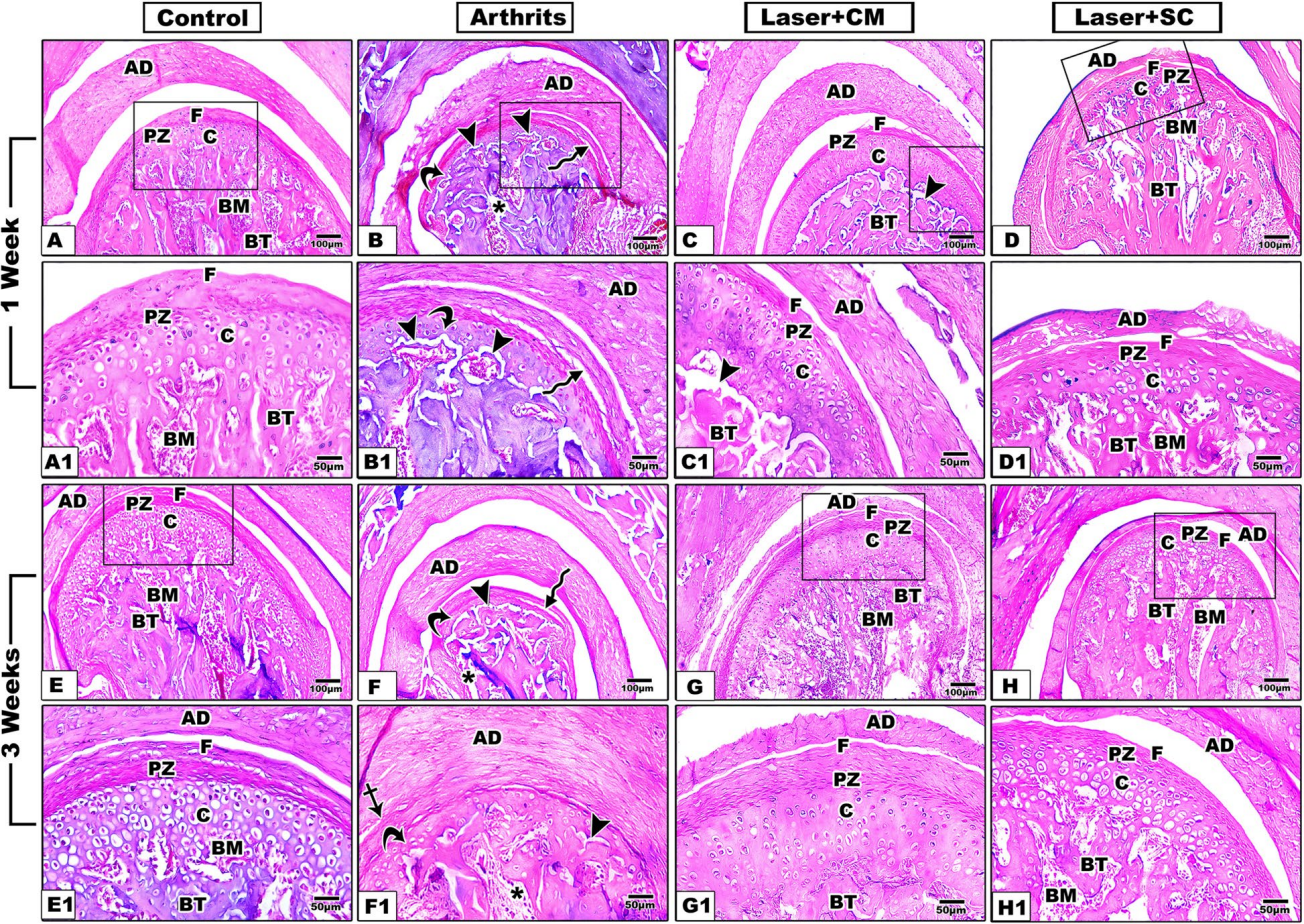
Hematoxylin and eosin staining of TMJ specimens of the control, arthritis, secretome/LLLT and ADSCs/LLLT groups at 1 and 3 weeks after treatment. AD: articular disc, F: fibrous zone, PZ: proliferative zone, C: condylar cartilage, BT: bone trabeculae, BM: bone marrow spaces, Irregular arrows indicate fibrous layer separation, Arrow heads indicates osteochondral interface detachment, Curved Arrows indicate cartilage atrophy, Crossed arrow indicates disc adhesion, Asterisks indicate widening of bone marrow spaces. (A-H): × 10; (A1-H1): × 20)

Group I (sham control), after one and three weeks of intervention, showed similar, normal histological structure where TMJ specimens showed normal articular disc, with regular collagen arrangement, separating the upper and lower joint compartments. The condylar head expressed three distinct zones: a regular, superficial, fibrous zone with some scattered chondrocytes, a proliferative zone, and a cartilage zone with well-arranged chondrocytes in their lacunae and nests. Normal osteochondral interface was observed overlying regularly arranged, dense trabecular subchondral bone (Fig. 2A, A1, E, E1).

In group II (arthritis) at week one, the articular disc showed marked thickening and fibrosis. The fibrous covering of the condyle was detached from the underlying tissues at some points. Marked atrophy of the condylar cartilage zone was observed and the underlying subchondral bone revealed some areas of resorption with concave howship’s lacunae (Fig. 2B, B1).

After three weeks, the articular disc showed more fibrosis and collagen disarrangement with interfibrous spacing and disc adhesion with complete disappearance of the lower joint compartment in some specimens. The proliferative zone of the condyle was almost diminished, and the condylar cartilage was much atrophied with hypocellularity and irregular arrangement of the chondrocytes. The cartilage bony interface was irregular, scalloped, and showed complete detachment in some specimens. Irregular trabeculation of the subchondral bone was noted (Fig. 2F, F1).

In group III (Secretome / LLLT) after one-week, regular outlines and collagen arrangement of the articular disc and fibrous layer were observed. In addition, the condyle gained much of its normal structure with a well-arranged cartilage layer. The articular disc was relatively thick, some areas of detachment between the condylar cartilage and the subchondral bone were detected, and the bone showed irregular trabeculation (Fig. 2C, C1). After three weeks of treatment, almost normal TMJ histology was restored with the normal structure of the articular disc. The condylar head restored the three distinct consecutive zones with normal thickness and structure overlying well-arranged subchondral bone (Fig. 2G, G1).

In group IV (ADSCs / LLLT) after one week, the disc appeared thinner, and the fibrous condylar layer was more regular compared to the arthritic group. The cartilage zone had more chondrocytes and showed normal osteochondral interface (Fig. 2D, D1). After three weeks, the disc thickness and collagen arrangement were more uniform, and the condylar head showed a distinct proliferative zone overlying a well-arranged hyaline cartilage and normal trabecular bone (Fig. 2H, H1).

### Articular disc thickness

**Fig. 3:**
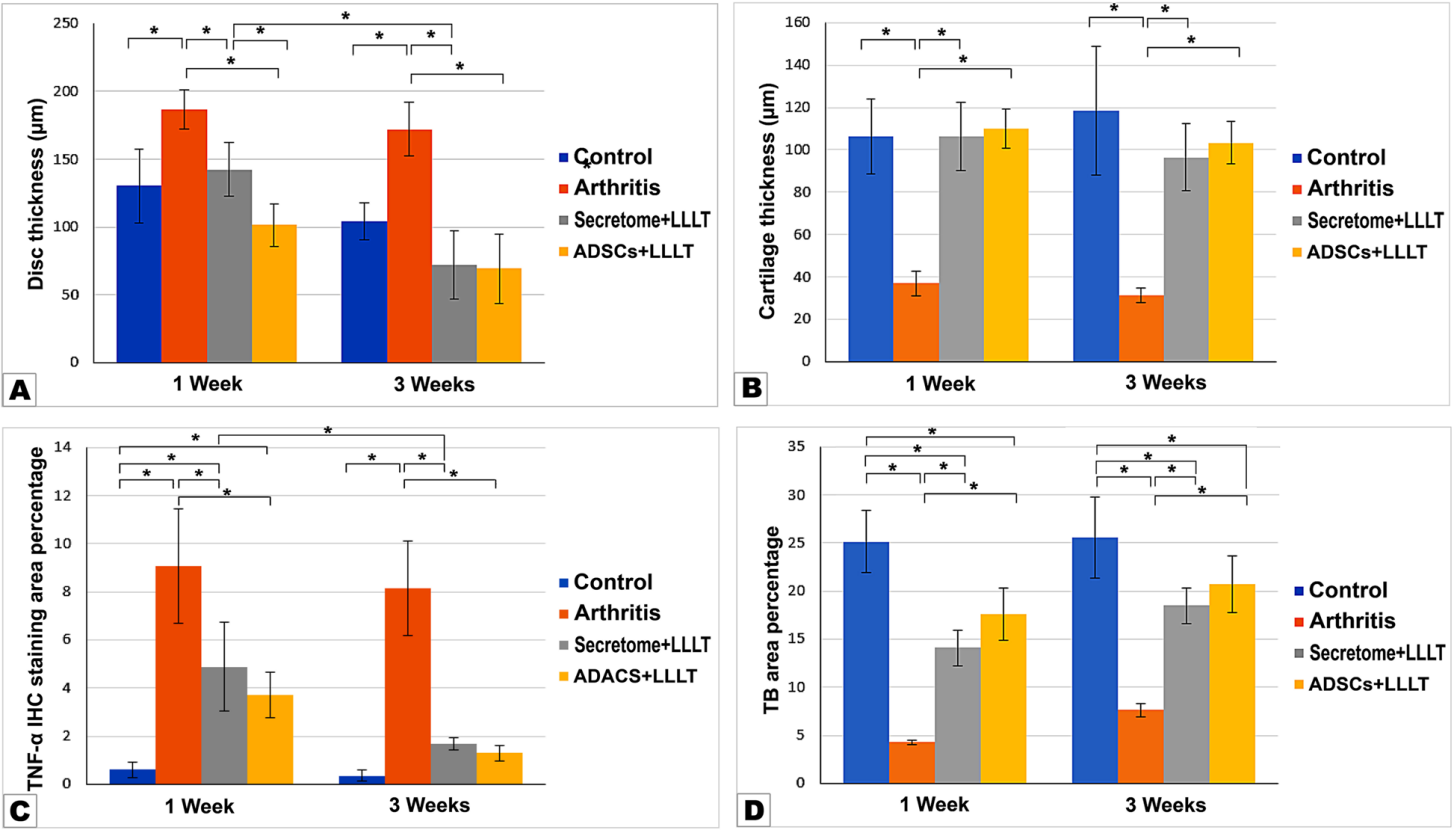
Bar graphs showing the statistical analysis results for: **A** Articular disc thickness, **B** Condylar cartilage thickness. **C** TNF-α immunohistochemical staining results. **D** Toluidine blue histochemical staining results. * Denotes statistical significance

Regarding the articular disc thickness, two-way ANOVA analysis revealed a significant interaction between the factors (intervention type and time) (F = 45.45, *P* < 0.0001,), as well as significant main effect of the intervention factor (F = 4.111, P = 0.0124) and time factor (F = 35.79, *P* < 0.0001). The Post Hoc Tukey test showed a significant increase in the articular disc thickness in the arthritis groups compared to the control group at 1 and 3 weeks. Interestingly, the treated groups (secretome/LLLT and ADSCs/LLLT) revealed a significant decrease compared to arthritis group and didn’t differ significantly than the control group at both time points. ADSCs/LLLT group showed significantly less disc thickening compared to the secretome/LLLT group one week after treatment, however after three weeks no significant difference was detected between them. In regard to time factor, Group III (secretome/LLLT) showed a significantly less disc thickness in the 3-weeks subgroup compared to 1 week subgroup (Table 2; Fig. 3A).

**Table 2:** Post Hoc Tukey test for pairwise comparison of factors affecting disc thickness by µm

**Time of assessment**	**Control**	**Arthritis**	**Secretome/LLLT**	**ADSCs/LLLT**
**1 Week**	130.39 ± 27.32	186.64 ± 14.31	142.60 ± 19.48	101.37 ± 15.30
		P1 = 0.0007^*^	**P1 = 0.9687** P2 = 0.0144^*^	**P1 = 0.2575** P2 < 0.0001^*^ P3 = 0.0268^*^
**3 Weeks**	104.15 ± 13.36	172.26 ± 19.81	72.11 ± 25.43	69.25 ± 25.61
	**P4 = 0.3775**	P1 < 0.0001^*^ **P4 = 0.927**	**P1 = 0.1597** P2 < 0.0001^*^ P4 < 0.0001^*^	**P1 = 0.0962** P2 < 0.0001^*^ **P3 > 0.9999** **P4 = 0.1577**

P Probability, ^*^significance < 0.05, P1: significance Vs control group, P2: significance Vs arthritis group, P3: significance Vs secretome/LLLT group, P4: Significance Vs 1 week group within the same intervention

### Condylar cartilage thickness

Two-way ANOVA analysis revealed a significant effect of interaction between intervention type and time factors (F = 65.62, *P* < 0.0001). However, neither intervention type (F = 1.229, *P* = 0.3116) nor time factors (F = 0.2940, *P* = 0.5907) had significant main effects.

Statistical analysis of the condylar cartilage thickness revealed a significant atrophy in the arthritis group at both 1 week and 3-week time points compared to control group. While both treated groups III and IV revealed a significant enhancement in cartilage thickness compared to the arthritis group at both time points and no significant difference than the control group. At both time intervals, ADSCs/LLLT group recorded better results than the secretome/LLLT group but didn’t reach significance level (Table 3; Fig. 3B).

**Table 3:** Post Hoc Tukey test for pairwise comparison of factors affecting cartilage thickness by µm

**Time of assessment**	**Control**	**Arthritis**	**Secretome + LLLT**	**ADSCs + LLLT**
**1 Week**	106.13 ± 17.68	36.93 ± 5.91	106.21 ± 16.01	110.24 ± 9.25
		P1 < 0.0001*	**P1 > 0.9999** P2 < 0.0001*	**P1 = 0.9998** P2 < 0.0001* **P3 = 0.9998**
**3 Weeks**	118.47 ± 30.17	31.41 ± 3.60	96.48 ± 15.77	103.35 ± 9.88
	**P4 = 0.8667**	P1 < 0.0001* **P4 = 0.9985**	**P1 = 0.2535** P2 < 0.0001* **P4 = 0.9581**	**P1 = 0.7035** P2 < 0.0001* **P3 = 0.9942** **P4 = 0.9941**

P Probability, ^*^significance < 0.05, P1: significance Vs control group, P2: significance Vs arthritis group, P3: significance Vs secretome/LLLT group, P4: Significance Vs 1 week group within the same intervention

### Immunohistochemical staining with TNF-α

**Fig. 4:**
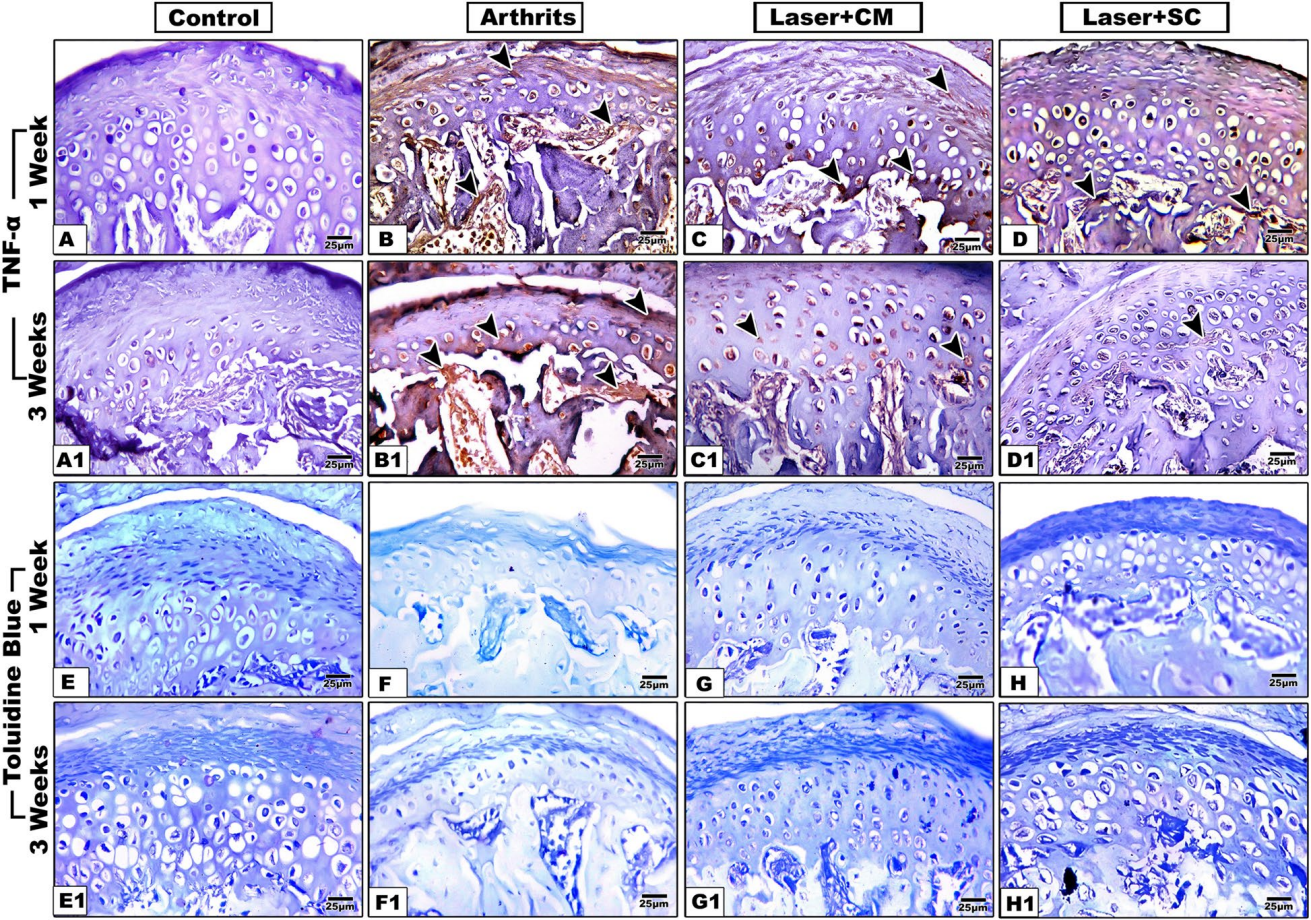
TNF-α and Toluidine blue staining of TMJ specimens of the control, arthritis, secretome/LLLT and ADSCs/LLLT groups at 1 and 3 weeks after treatment. Arrow heads show positively stained cells (× 40)

Positive immunostaining appeared as brown deposits in the different layers of the condylar head. For the control group, minimal reaction was detected, while variable degrees of inflammation were recorded in the other groups with the highest inflammatory reaction observed in the arthritis group followed by the secretome/LLLT, then ADSCs/LLLT groups (Fig. 4A-D, A1-D1 × 40). Two-way ANOVA analysis revealed a significant effect of intervention factor (F = 3.099, *P* = 0.0374), time factor (F = 19.72, *P* < 0.0001) and intervention-by-time interaction (F = 81.64, *P* < 0.0001).

The analysis showed a significantly increased inflammatory reaction in the arthritis group at both time points compared to control group. Also, the treated groups III and IV revealed a significant increase compared to the control group one week after treatment. However, they showed significantly decreased inflammation compared to the arthritis group through the whole experiment and after three weeks they didn’t differ significantly than the control group. Regarding time, the secretome/LLLT treated group showed significantly less inflammation in the 3-week subgroup compared to the 1-week subgroup (Table 4; Fig. 3C).

**Table 4:** Post Hoc Tukey test for pairwise comparison of factors affecting TNF-α Immunohistochemical staining percentage area

**Time of assessment**	**Control**	**Arthritis**	**Secretome + LLLT**	**ADSC + LLLT**
**1 Week**	0.61 ± 0.33	9.08 ± 2.37	4.88 ± 1.85	3.72 ± 0.96
		P1 < 0.0001^*^	P1 < 0.0001^*^ P2 < 0.0001^*^	P1 = 0.0051^*^ P2 < 0.0001^*^ **P3 = 0.7909**
**3 Weeks**	0.35 ± 0.22	8.14 ± 1.96	1.68 ± 0.24	1.29 ± 0.32
	**P4 > 0.9999**	P1 < 0.0001^*^ **P4 = 0.9222**	**P1 = 0.6735** P2 < 0.0001^*^ P4 = 0.0035^*^	**P1 = 0.9202** P2 < 0.0001^*^ **P3 = 0.9996** **P4 = 0.0552**

P Probability, ^*^significance < 0.05, P1: significance Vs control group, P2: significance Vs arthritis group, P3: significance Vs secretome/LLLT group, P4: Significance Vs 1 week group within the same intervention

### Toluidine blue staining

Toluidine blue staining was applied to metachromatically stain the proteoglycan content of the articular cartilage (Fig. 4E-H, E1-H1 × 40). Two-way ANOVA analysis revealed a significant interaction between factors (F = 121.2, *P* < 0.0001), significant main effect of time (F = 14.53, *P* = 0.0005), but insignificant main effect of intervention type factor (F = 1.326, *P* = 0.2794).

The post-hoc pairwise comparison revealed a significant decrease in cartilage proteoglycan content in all groups compared to the control group with ADSCs/LLLT group expressed the highest values, followed by the secretome/LLLT group and the arthritis group respectively. However, the treated groups revealed a significantly higher content compared to arthritis group at both time points (Table 5; Fig. 3D).

**Table 5:** Post Hoc Tukey test for pairwise comparison of factors affecting toluidine blue staining percentage area

**Time of assessment**	**Control**	**Arthritis**	**Secretome + LLLT**	**ADSCs + LLLT**
**1 Week**	25.16 ± 3.22	4.29 ± 0.25	14.11 ± 1.85	17.57 ± 2.70
		P1 < 0.0001^*^	P1 < 0.0001^*^ P2 < 0.0001^*^	P1 = 0.0002^*^ P2 < 0.0001^*^ **P3 = 0.2924**
**3 Week**	25.55 ± 4.26	7.62 ± 0.65	18.47 ± 1.86	20.71 ± 2.96
	**P4 > 0.9999**	P1 < 0.0001^*^ **P4 = 0.3392**	P1 = 0.0005^*^ P2 < 0.0001^*^ **P4 = 0.0871**	P1 = 0.0396^*^ P2 < 0.0001^*^ **P3 = 0.7877** **P4 = 0.4104**

P Probability, ^*^significance < 0.05, P1: significance Vs control group, P2: significance Vs arthritis group, P3: significance Vs secretome/LLLT group, P4: Significance Vs 1 week group within the same intervention

## Discussion

Arthritis is a debilitating, challenging disorder that imposes psychological and economic burdens on individuals. Many treatment modalities have been implicated; however, none of the conventional therapies has completely succeeded to arrest the progression of the disease or reverse the structural changes of cartilage and bone [20]. Complete Freund’s adjuvant induced arthritis rat model, which depends on injection of inactivated Mycobacterium tuberculosis bacteria, is a widely used inflammatory model in pathogenic investigations and molecular studies due to its similarity to human rheumatoid arthritis pathophysiologically and pharmacologically [21].

Application of LLLT in treatment of TMJ arthritis has gained wide interest as a novel treatment where many studies showed the biomodulatory influence of LLLT with different parameters on arthritic joints as it induces tissue regeneration and wound healing by immunomodulation and regulating inflammatory mediators [22, 23]. In a previous study, we managed to test the efficacy of LLLT on induced TMJ arthritis in rats, and the results showed significant amelioration of arthritic changes. However, complete restoration of the normal histological architecture was not achieved [16].

So, the current study aimed to exploit the proven biostimulatory effects of LLLT to enhance the therapeutic potentials of ADSCs or their derived secretome. The findings revealed the significant comparable efficacy of both treatments associated with LLLT to enhance TMJ regeneration.

In the current study, the arthritis groups showed severe changes in the histology of the TMJs which were more aggravated at the third week. This coincides with Adaes et al. [24] who reported that manifestation of cartilage damage and arthritic pathological changes in osteoarthritic models were more expressed between the second and fourth weeks.

The findings of the current study also agreed with Kalladka et al. [25] who stated that TMJ osteoarthritis causes degeneration of TMJ soft and hard tissues leading to cartilage abrasion and subchondral bone resorption. Similar to our results, induced arthritis in rat models was found to cause degeneration and atrophy of the articular cartilage, subchondral bone resorption, collagen disarrangement, and increased articular disc thickness [7, 10, 22]. Moreover, in accordance with the current results, Ishizuka et al. [26] reported atrophy of the chondrogenic proliferative layer of the TMJ condyle in arthritic mice.

TMJ specimens in group III (Secretome / LLLT) showed regular histology and arrangement of the articular disc and the condylar head. This result can be attributed to the fact that ADSCs secretome was found to contain multiple soluble factors that affect tissue healing such as hepatocyte growth factors (HGF), transforming growth factor (TGF)-β1, insulin-like growth factor 1, fibroblast growth factor (bFGF), and vascular endothelial growth factor (VEGF) [27]. Zheng et al. [28] owed the therapeutic effect of MSC secretome, particularly, to the extracellular vesicles (EVs), which can maintain controlled environment and keep their content integrity due to their double layer structure.

To the best of our knowledge, no studies combined MSC secretome with LLLT for treating TMJ arthritis, but secretome alone has been applied with promising results, where Cheng et al. [29] reported improvement in the articular cartilage and less defective cartilage matrix and chondrocytes in the ADSCs conditioned media (CM) -treated groups in osteoarthritic rat knees. In another study conducted by Nazemian et al. [30], intraarticular injection of MSC-CM was able to decrease edema, hyperalgesia, serum TNF-α levels and intracellular signaling pathway factors activity alleviating inflammatory reactions of acute and chronic phases of CFA-induced arthritis. Yew, et al. [31] also reported that treatment with MSC-CM managed to reduce TNF-α, spinal nuclear factor kappa B (NF-κB), p38 mitogen-activated protein kinase (P38MAPK) expression and interferon gamma (IFN-γ) release, and increase anti-inflammatory cytokines secretion at the site of inflammation.

Concomitantly, other studies also demonstrated the beneficial effects of secretomes derived from bone marrow stem cells (BMSCs) and umbilical cord mesenchymal stem cells (UCSCs) via histological staining, showing reduced cartilage destruction, better microarchitecture of subchondral bone, and enhanced deposition of cartilage matrix. Secretome application seemed to have a protective effect by preventing chondrocyte apoptosis, reducing extracellular matrix (ECM) proteolysis, repairing the damaged cartilage with hyaline cartilage instead of fibrocartilaginous tissue, as well as reducing osteocyte apoptosis and osteoclastic activity [32, 33]. MSC exosomes have also revealed promising results in osteochondral regeneration of arthritic defects [34, 35]. On the other hand, secretome may contain different components that can cause adverse effects, and more proteomic analysis and studies are required to unravel its exact constituents [36].

Combination of ADSCs therapy with LLLT in group IV also resulted in significant regeneration of the joint structure where MSC are known to mediate tissue regeneration by different mechanisms. Molnar et al. [37] attributed the therapeutic effect of MSC therapy in osteoarthritis primarily to the immunomodulatory and trophic effects, and concluded that either autocrine or paracrine effects enhanced the angiogenesis and mitosis while preventing apoptosis and excessive tissue fibrosis. Therefore, ADSCs were reported to induce the regeneration of arthritic knees by reducing the expression of matrix metallopeptidase 13 (MMP13) and Collagen X and increasing Collagen II [38]. Moreover, clinically, ADSCs were found to alleviate TMJ osteoarthritis as confirmed by significant reduction in transforming growth factor beta 1 (TGFβ1) levels in patients’ synovial fluids, together with better clinical examination results [39].

Photobiomodulation was reported to induce stem cell migration and proliferation promoting tissue regeneration [40]. It was also demonstrated to enhance the chondrogenic potential of ADSCs [41]. In a similar study, combining ADSCs with photobiomodulation in knee arthritic models significantly ameliorated tissue regeneration through reduction of proinflammatory regulators and upregulation of tissue inhibitors of metalloproteinase (TIMP) and collagen 2 (COL 2) expression in the laser associated groups more than the groups treated with ADSCs only or laser only [42]. In the same context, application of LLLT together with BMSCs for treatment of induced knee joint osteoarthritis in rabbits was found to enhance bone formation and reduce inflammation [43]. Additionally, ADSCs along with LLLT therapy was reported to promote bone regeneration in femoral and calvarial defects [44, 45].

However, in some reported studies, laser biostimulation for TMJ disorders didn’t show significant effects histologically in experimental animals [46] or in human clinical studies regarding pain and TMJ symptoms [47]. This may be justified by the very wide variations in the type of applied laser therapy and parameters between different studies.

Moreover, cell-based therapy carries many risks which can’t be ignored. According to a study conducted by Zhang et al. [48], MSC injection for treating cartilage defects resulted in significant immune rejection in rats without neonatal desensitization. Nori et al. [49] also reported tumor formation after pluripotent stem cells transplantation into a spinal cord injury mouse model. Such drawbacks encouraged scientists to explore the therapeutic potentials of stem cell secretomes rather than whole cells. However Conforti et al. [50] reported that secretome alone has failed to exert the required immunomodulatory effects of native cells, and explained that cell to cell contact was needed to regulate lymphocyte function and proliferation.

In the current study TNF-α was used as an inflammatory marker, where TNF-α is a cytokine that is related to inflammation and tissue degeneration in arthritis [11]. The inflammatory reaction was more expressed in the first week than the third week which agrees with Adaes et al., [24] who reported that the inflammatory phase of osteoarthritis was mostly pronounced at the first week.

In addition, ADSCs and secretome treatments associated with LLLT in our study showed significant reduction in the inflammatory response of arthritic joints as measured by TNF-α immunohistochemical reaction. This coincides with Carvalho et al. [51] who reported marked anti-inflammatory effect of LLLT in arthritic rat TMJs. Corroborating these results, Pires et al. [52] tested the anti-inflammatory effect of LLLT by real-time PCR and detected reduced expression of interleukin IL-6, cyclo-oxygenase 2 (COX-2), TGF-β and TNF-α in a rat tendinitis model.

In a study by Stancker et al. [42] treatment with ADSCs together with LLLT resulted in significant decrease of proinflammatory cytokines as IL-1, IL-6, and TNF-α in rat arthritic knee models. Cheng et al. [29] also reported less inflammation in rat arthritic knee joints treated with ADSCs- secretrome as proved by reduction of terminal deoxynucleotidyl transferase dUTP nick end labeling (TUNEL), IL-1, and MMP-13.

In a previous study, we managed to compare the effect of ADSCs and ADSCs derived secretome on the healing of mucosal tongue defects and the results showed no significant difference in the inflammatory response or the proliferative cell capacity between the two groups [53]. However, Rani et al. [54] postulated that MSC secretome elucidates less inflammatory response than complete cells attributed to the less concentration of immune complexes in the secretome when compared to whole cells making secretome a possibly safer therapy.

Toluidine blue staining was used to assess the proteoglycan content where darker staining or metachromasia indicates more proteoglycans [11, 55]. Similar to our findings, Lemos et al. [22] reported loss of the proteoglycans in arthritic TMJs as expressed by less metachromasia of the condylar cartilage in histological slides. While after LLLT, more proteoglycan was detected indicating cartilage regeneration. Concomitantly, SHED [56] and BMSCs [57] secretome treatment in arthritic TMJ and knee joints respectively showed less cartilage depletion and increased proteoglycans. Kehoe et al. [11] also reported the beneficial effect of MSC intra-articular injection in the knee joints as expressed by more proteoglycan content stained by toluidine blue.

In the present study both the ADSCs and the secretome treatments associated with LLLT expressed high therapeutic potentials for TMJ arthritis with no significant difference in their results even though the ADSCs/LLLT group showed relatively less inflammatory response and higher proteoglycan content. In corroboration with our results, some studies compared the therapeutic influence of MSC and their secretome in treating joint arthritis and reported comparable results with no significant differences between them [36, 57]. Similarly both ADSCs and their secretome were equally effective in treating induced tongue ulcers in rats [53]. On the other hand, other studies reported different results, where Gabr et al. [58] detected better effect of MSC on renal failure models than their secretome, while Abdel Aal et al. [59] showed higher influence of secretome treatment than MSC in liver injury model.

Therefore, stem cells possess high therapeutic potential and some advantages over cell free therapy such as the differentiation capacity, besides their homing ability [60]. On the other side, secretome treatment can reproduce the effects achieved by cell therapy with the benefit of eliminating problems associated with cell transplantation as difficulty in harvesting autologous cells, controlling differentiation of the transplanted cells and immunogenicity. However, there are still some limitations that need to be overcome such as the wide range of different unknown secretome components, the need for standardization of secretome production, the variable factors affecting composition like passage number and culture conditions [61]. All these issues require further investigations and research to be performed before stem cell secretome can be widely applied as an efficient therapy for TMJ arthritis.

## Conclusion

LLLT possesses a good biostimulatory cellular effect and associated with ADSCs or their derived secretome, it can efficiently alleviate TMJ arthritis through enhancing the anti-inflammatory effect, inducing cartilage repair, trabecular bone rearrangement, and articular disc restoration.

## Data Availability

All data generated or analyzed during this study are included in this published article.
